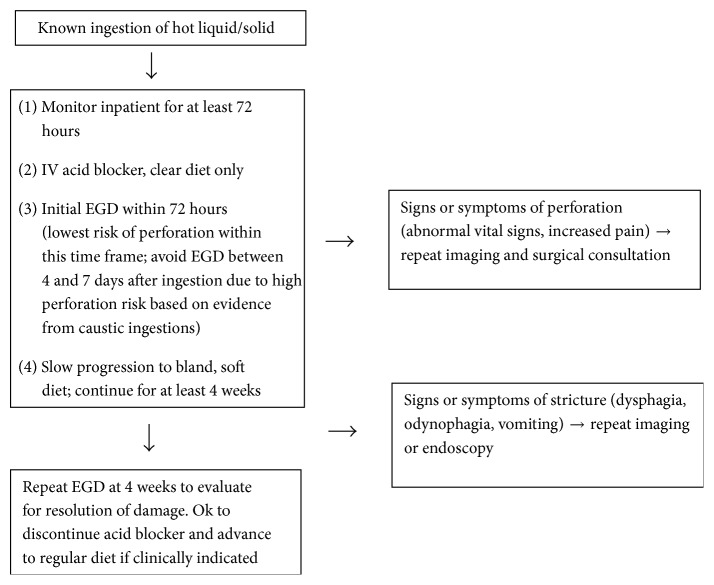# Thermal Esophageal Injury following Ingestion of Boiling Mushroom Water

**DOI:** 10.1155/2017/1859352

**Published:** 2017-04-18

**Authors:** Allison Prevost, Adam Talley, Emily Klepper, Elizabeth McDonough

**Affiliations:** ^1^Pediatric Residency Program, Our Lady of the Lake Children's Hospital, Baton Rouge, LA, USA; ^2^Louisiana State University Health Sciences Center, New Orleans, LA, USA; ^3^Our Lady of the Lake Children's Hospital, Baton Rouge, LA, USA

## Abstract

Thermal esophageal and gastric damage from ingestion of hot liquids is poorly studied in pediatrics. Limited case reports exist in the literature. Many cases presented with chest pain, dysphagia, and odynophagia. Variable histologic findings were reported. No definitive management guidelines exist for such injuries. We provide a report of the acute assessment and management of an obvious thermal esophageal injury and contribute to what is known about this presentation. A 16-year-old male presented with odynophagia, dysphagia, and hematemesis following ingestion of “nearly boiling” mushroom water. Ondansetron, pantoprazole, ketorolac, maintenance intravenous fluids, and a clear liquid diet were started. At sixty hours after ingestion, an esophagogastroduodenoscopy (EGD) revealed blistering and edema of the soft palate and epiglottis, circumferential erythema of the entire esophagus with an exudate likely to be desquamated mucosa, and linear erythema of the body and fundus of the stomach. An EGD one month after ingestion showed no residual effects from the injury. The pantoprazole was weaned and restrictions to his diet were lifted. To better standardize care in these rare esophageal injuries, the development of a clinical care algorithm may be beneficial to provide clinicians with a guide for management based on outcomes of previously reported cases.

## 1. Case Presentation

**Figure 1:**
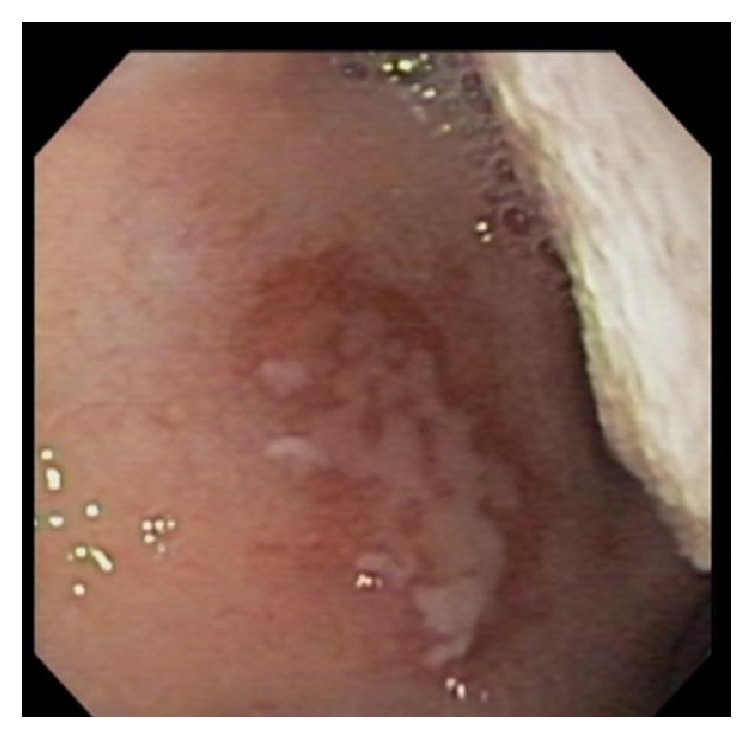
Blister on palate.

**Figure 2:**
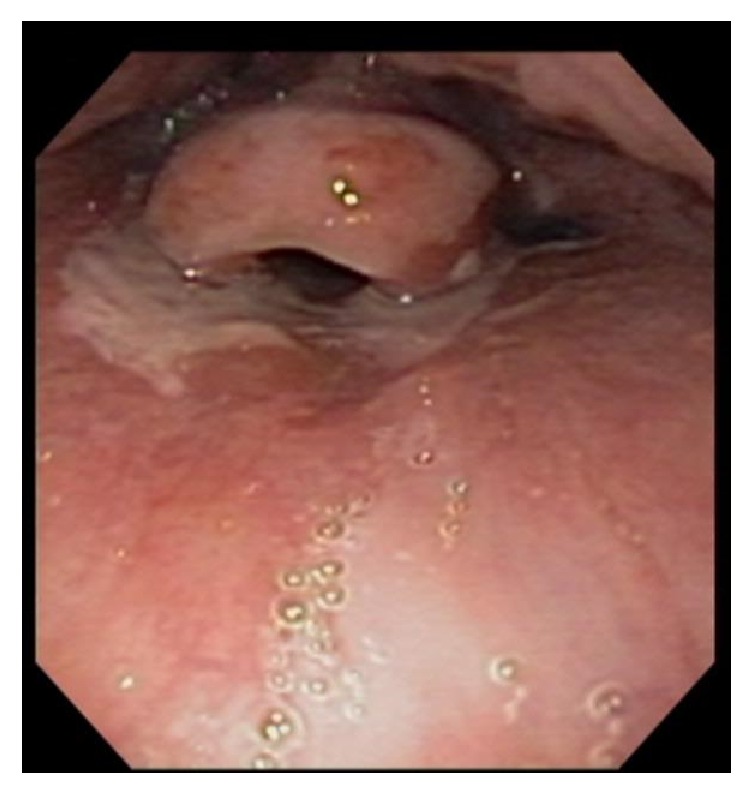
Edema and blisters on epiglottis.

**Figure 3:**
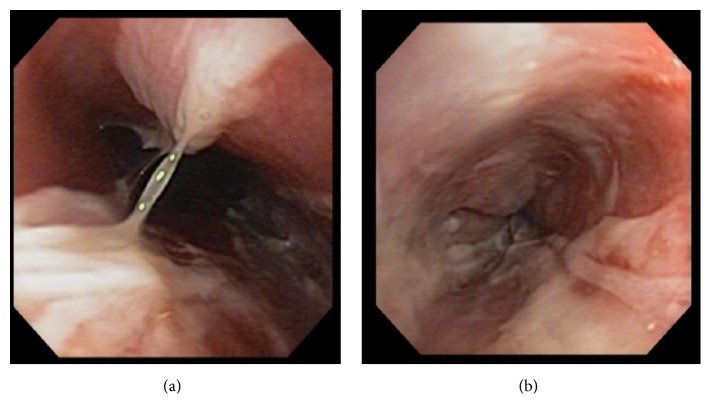
Erythema and desquamation of esophagus.

**Figure 4:**
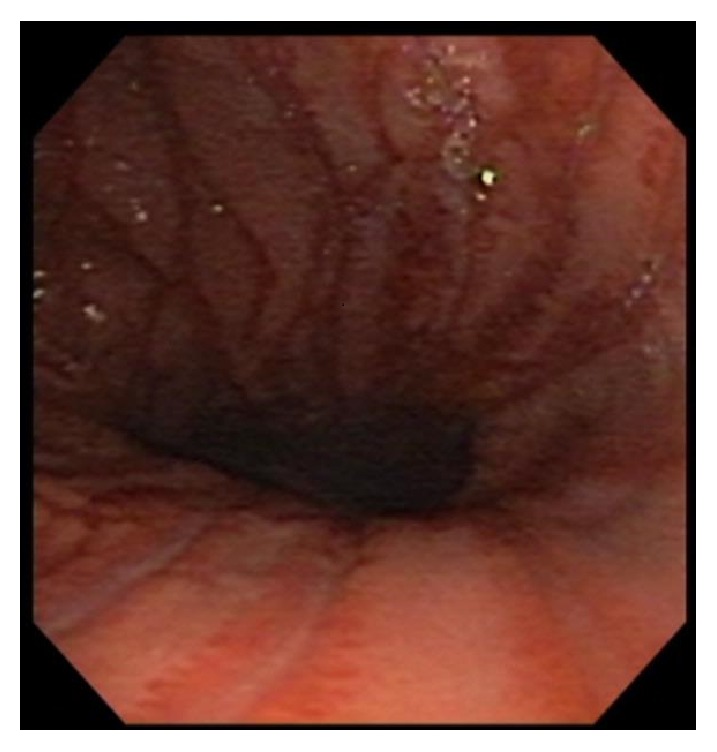
Linear erythema of gastric body and fundus.

**Figure 5:**
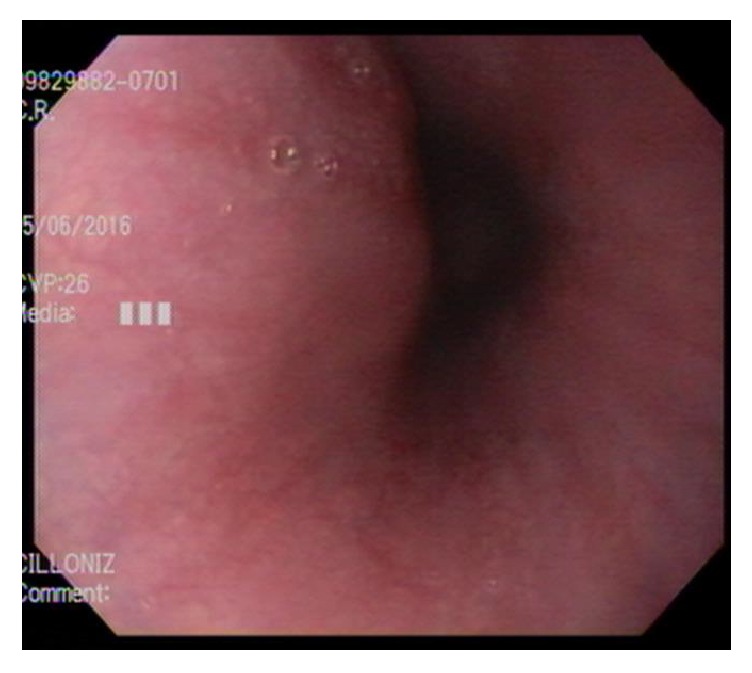
Erythema and desquamation of esophagus.

A 16-year-old male presented to the emergency department complaining of odynophagia, dysphagia, and bloody emesis since ingestion of “nearly boiling” mushroom tea. The patient reported picking mushrooms from his yard and adding them to microwave-heated water with the intent to make a tea for recreational intoxication. He quickly drank a 10–12-ounce gulp of the tea in an attempt to hide it from his approaching mother. Immediately, he regurgitated the liquid and began having throat pain and hematemesis. Twenty hours after ingestion, in the emergency department, a chest X-ray and esophagram were within normal limits and the patient was admitted to the hospital pediatric service in stable condition. The patient was started on intravenous (IV) ondansetron, pantoprazole, ketorolac (as needed), maintenance IV fluids, and a clear liquid diet. At sixty hours after ingestion, an esophagogastroduodenoscopy (EGD) was performed which revealed blistering and edema of the soft palate (Figure 1) and epiglottis (Figure 2), diffuse and circumferential erythema of the entire esophagus with an exudate likely to be desquamated mucosa (Figures 3(a) and 3(b)), and linear erythema of the body and fundus of the stomach (Figure 4). The patient was evaluated by otolaryngologist who performed a laryngoscopy and reassured that his risk for airway damage and inflammation was minimal. Additionally, Cardiothoracic Surgery was consulted and recommended a computed tomography (CT) scan of the thorax with IV contrast due to concern for perforation causing mediastinitis. The CT scan showed no abnormalities. At this time an oral sucralfate suspension and an oral rinse containing magnesium hydroxide, viscous lidocaine, and diphenhydramine were started for further symptomatic treatment, and peripheral parenteral nutrition was initiated due to poor oral intake. Three days after ingestion, the patient tolerated sports drinks and popsicles without the need of pain medication. The following day he tolerated grits and a smoothie so the parenteral nutrition was discontinued. Finally, five days after ingestion the patient continued to show improvement and was discharged with orders to continue a soft diet for two weeks while taking oral pantoprazole daily and sucralfate every six hours. At two-week follow-up with gastroenterology, the patient reported continued improvement and denied pain or difficulty swallowing. A repeat esophagram to evaluate for stricture was normal. One month after ingestion, a repeat EGD was performed which was completely normal and showed no residual effects from the previous thermal injury (Figure 5). At this time the pantoprazole was weaned and restrictions to his diet were lifted. The patient has done well with no need for further intervention or follow-up.

## 2. Discussion

Thermal esophageal and gastric damage due to ingestion of hot liquids is not very well studied or reported, especially in pediatrics. We were able to find fourteen case reports of thermal esophageal injuries, none of the patients being under 20 years of age. However, this is likely an underestimate because most cases are most likely unreported. All were accidental ingestions of hot food or liquid, and the majority of cases presented with symptoms of chest pain, dysphagia, and odynophagia. In several reports the cause of the symptoms was obvious at presentation, but some required a thorough recall of recent ingestions. Our case provides a complete report of the acute assessment and management of an obvious thermal esophageal injury and may contribute to what is known about this unique presentation.

Most cases cited thus far describe a pseudomembrane appearance to the esophageal mucosa on initial endoscopy. This appears as a thin, white tissue layer over affected areas and likely represents sloughing of the mucosa or ruptured bullae. The pseudomembrane phenomenon has been described from as early as day 0 after ingestion to as late as 4 weeks after ingestion [1, 2]. Some studies also describe a “candy-cane” appearance to the esophageal mucosa. It is possible that this finding is associated with a specific stage in the healing process rather than the direct injury itself [2, 3]. Lee et al. describes a 45-year-old woman who ingested hot tea and initially was found to have pseudomembrane damage at day 7 endoscopy which progressed to candy-cane damage seen on day 14 [2]. In another scenario, Choi et al. describe a 38-year-old woman who drank hot tea and was found to have pseudomembrane on day 8 and candy-cane appearance on day 15 [3]. However, Cohen and Kegel describe a candy-cane appearance to the distal esophagus just 2 days after ingestion of boiling water while freebasing cocaine, though that particular situation is unique [4]. It is also notable that all cases describing ingestion of a hot solid found localized or longitudinal blisters or ulcers in the esophageal mucosa, whereas ingestion of hot liquids revealed diffuse, circumferential esophageal damage, likely due to the flow of liquid down the esophagus [5, 6].

All but one of the cases responded to conservative treatment and resolved without sequelae. Kitajima et al. described the unique case of a 28-year-old Japanese man whose thermal esophageal burns from drinking hot coffee led to an esophageal stricture. This man initially presented on the day of ingestion with such extensive edema of his pharynx that his airway became compromised and endoscopy was impossible. After 40 days of conservative management and parenteral nutrition, esophagoscopy revealed healing of the mucosal edematous changes. He was then discharged and cleared to resume oral intake. After discharge he gradually began experiencing symptoms of dysphagia until, at 5 months after ingestion, he was found to have a pin-hole stricture of his esophagus. The stricture was then successfully treated with esophagectomy and ileocolon interposition [7]. In contrast to this report, other cases, including our own, showed resolution of burns on follow-up endoscopy and did not result in long-term complications [1–6, 8, 9]. For the majority of thermal esophageal burns, proton pump inhibitor [2, 3, 6, 8], sucralfate [2], and a slow progression of oral intake appear to be sufficient for resolution [8].

Compared to thermal injuries, caustic injuries of the esophagus with acidic or alkaline substances are reported much more frequently and extensively in medical literature. These ingestions are typically accidental in children and associated with suicidal intentions in adolescents and adults. The endoscopic appearance is variable depending on the type of substance ingested but may appear similar to that seen with thermal burns [8]. Endoscopy to assess severity has shown to be important in the prognosis of caustic burns. Grade 1 mucosal lesions are unlikely to result in stricture or perforation while more severe Grade 3 lesions have a higher likelihood. Overall the chance of caustic ingestion resulting in a stricture is between 10 and 20 percent. In addition, the greatest risk of a perforation occurring is between days 5 and 15 after ingestion due to the presence of weak granulation tissue and the absence of strong collagen fibers during this time period [9]. Speculating from the results of the cases we found, thermal esophageal injuries appear less likely than caustic injuries to result in strictures or other sequelae.

Without extensive studies on the varying presentations and complications of thermal esophageal injuries, a conservative management approach must be taken. Any acute change in patient status during the first week of healing must be noted and monitored closely. Also clinicians should be aware of the small but legitimate risk of the patient eventually developing a stricture. In order to better standardize patient care in these rare esophageal injuries, the development of a clinical care algorithm may be beneficial. This could provide clinicians with a guide for management based on the outcomes of previously reported cases. Our suggested approach is presented in Figure 6.

## Figures and Tables

**Figure 6:**